# The complete mitochondrial genome of Indian Cuckoo *Cuculus micropterus* (Aves: Cuculiformes)

**DOI:** 10.1080/23802359.2021.1959461

**Published:** 2021-08-02

**Authors:** Yu’ang Tian, Jingtong Xu, Liang Dou, Anqin Zheng, Lu Qiao, Jiqin Xie, Xiuyue Zhang

**Affiliations:** a Key Laboratory of Bio-resources and Eco-environment, Ministry of Education, College of Life Sciences, Sichuan University, Chengdu, China; b Sichuan Key Laboratory of Conservation Biology on Endangered Wildlife, College of Life Sciences, Sichuan University, Chengdu, PR China

**Keywords:** *Cuculus micropterus*, complete mitochondrial genome, gene arrangement

## Abstract

The Indian Cuckoo, *Cuculus micropterus*, belongs to the family Cuculidae. In this paper, we sequenced and analysized the complete mitochondrial genome of *C. micropterus*. The complete mitochondrial genome of *C. micropterus* is 17,541 bp in length, which was submitted to the NCBI database under the accession number MZ048030. It contains 13 protein-coding genes, 22 transfer RNA genes, two ribosome RNA genes, and one non-coding control region. The overall base composition of the mitochondrial DNA is 33.2% for A, 24.2% for T, 29.8% for C, and 12.8% for G, with a GC content of 42.6%. In order to explore the molecular phylogenetics evolution of Cuculidae, the nucleotide sequence data of 13 PCGs of *C. micropterus* and other 11 Cuculiformes were used for the phylogenetic analysis. The result shows that *C. micropterus* is closely related to *Cuculus canorus bakeri*. The study contributes to illuminating the taxonomic status of *C. micropterus*, and may facilitate further investigation of the evolution of Cuculidae.

Indian Cuckoo (*Cuculus micropterus* Gould, 1838) is a grayish and medium-size bird within the family Cuculidae. It is characterized by long, pointed wings, whose margin is all white without any spot. Its lower body looks white starting from the lower chest, mixed with black transverse spots. The main characteristic is its gray tail, with broad sub-terminal black bands and a white tip (Zhou and Liang 2016). These birds are mainly distributed in Bangladesh, Brunei, Cambodia and China. The most special thing of *C. micropterus* is their loud sound, which consists of four notes and can be transcribed as ‘bo-ko-ta-ko’. Besides, Cuckoos are alert and secretive. As a result, their cries are often heard by people but there are few witnesses (Wang 2012). Instead of nesting and feeding offspring in person, the species lays their eggs in the nests of the host, Azure-winged Magpie (*Cyanopica cyanus*) (Zhang et al. 2017). Acoording to the ICUN’s 2018 Red List of Least Concern Species (LC), it is listed as one of the least concern species (IUCN 2018). The complete mitochondrial genome is commonly used to analyze the genetic relationship and evolutionary status of species. However, the complete mitochondrial genome of *C. micropterus* has not been reported yet. Therefore, we conducted a complete sequencing of the mitochondrial genome of *C. micropterus.*

The specimen was collected from an accidental dead *C. micropterus*, which was found at Aba Hongyuan Airport, Sichuan Province (Latitude: 32°31′57.50′′N, Longtitude:102°21′43.02′′E) in September 2018. It is now preserved at the Sichuan Key Laboratory of Conservation Biology on Endangered Wildlife (College of Life Sciences, Sichuan University, Liang Dou, 547738389@qq.com) under the voucher number HY091601. The total genomic DNA was extracted from the muscle tissue with the TIANamp Genomic DNA kit (TIANGEN, Beijing, China) according to the manufacturer's instructions. The complete mitochondrial genome was amplified from 13 overlapping fragments by PCR and the primers were designed from known mitochondrial genomes of *Cuculus poliocephalus* (NC028414). The obtained portions of the mitogenome were then used to design species-specific primers by Primer Premier 5.0 to link the overlapping fragments. The PCR produces were sequenced by ABI PRISM 3730 DNA sequencer and the software DNA SeqMan was used for sequences assembly.

The complete mitochondrial genome of *C. micropterus* is a circular DNA molecule, which is 17,541 bp in length and has been submitted to the NCBI database under the accession number MZ048030. The full genome of *C. micropterus* mitochondrial contains 13 protein-coding genes (PCGs), two ribosomal RNA genes (12S rRNA and 16S rRNA), 22 transfer RNA genes (tRNA), and 1 non-coding control region (D-loop). The arrangement of the multiple genes is similar to other Cuculidae species. Most genes are transcribed on the heavy (H) strand, while the ND6 and other eight tRNA genes (tRNA-Gln, tRNA-Ala, tRNA-Asn, tRNA-Cys, tRNA-Tyr, tRNA-Ser, tRNA-Glu, and tRNA-Pro) are transcribed on the light (L) strand. The overall base composition is 33.2% for A, 24.2% for T, 29.8% for C, and 12.8% for G, with a GC ratio of 42.6%.

Eleven-thirteenths of PCGs use ATG as the start codon, while COX1 and ND3 select GTG and ATA, respectively. The PCGs have four types of termination codon, including TAA for COX2, ND3, ND4L, ND6, ATP6, ATP8 and Cytb; AGA for ND1 and ND5; AGG for COX1; and an incomplete stop codon T for ND2, ND4, and COX3. It is found that some birds have an extra single ‘C’ insertion in ND3 (Mindell et al. 1998; Yan et al. 2017). However, the base in *C. micropterus* is replaced by a single ‘A’, which is consistent with the result of some other species of Cuculidae like *Cuculus canorus bakeri* (Qiu et al. 2019), *C. poliocephalus* (Wang et al. 2016) and *Eudynamys taitensis* (Pratt et al. 2009). It is also possible that these birds can still use the programmed translational frame shifting (Russell and Beckenbach 2008) to tolerate the ‘A’ frame shift insertions. Unlike these mitochondrial genomes of many other birds, which have a pseudo-control region (Song et al. 2015), *C. micropterus* has only one single control region while overlapping bases and noncoding bases are also found in *C. micropterus* mitochondrial genome.

In order to explore the molecular phylogenetics and evolution of Cuculidae, the phylogenetic analysis is conducted based on the nucleotide sequence data of 13 PCGs of *C. micropterus* and other 11 Cuculiformes species. *Caprimulgus indicus* was selected as the outgroup. The tree constructed by Bayesian phylogenetic analysis is shown in the Figure 1. The Bayesian analysis suggests that the *C. micropterus* is closely related to the *C. canorus bakeri*. The complete mitochondrial genome of *C. micropterus* will contribute to the species delimitation, phylogenetic analysis of the family Cuculidae and other relative studies in the future.

**Figure 1.**
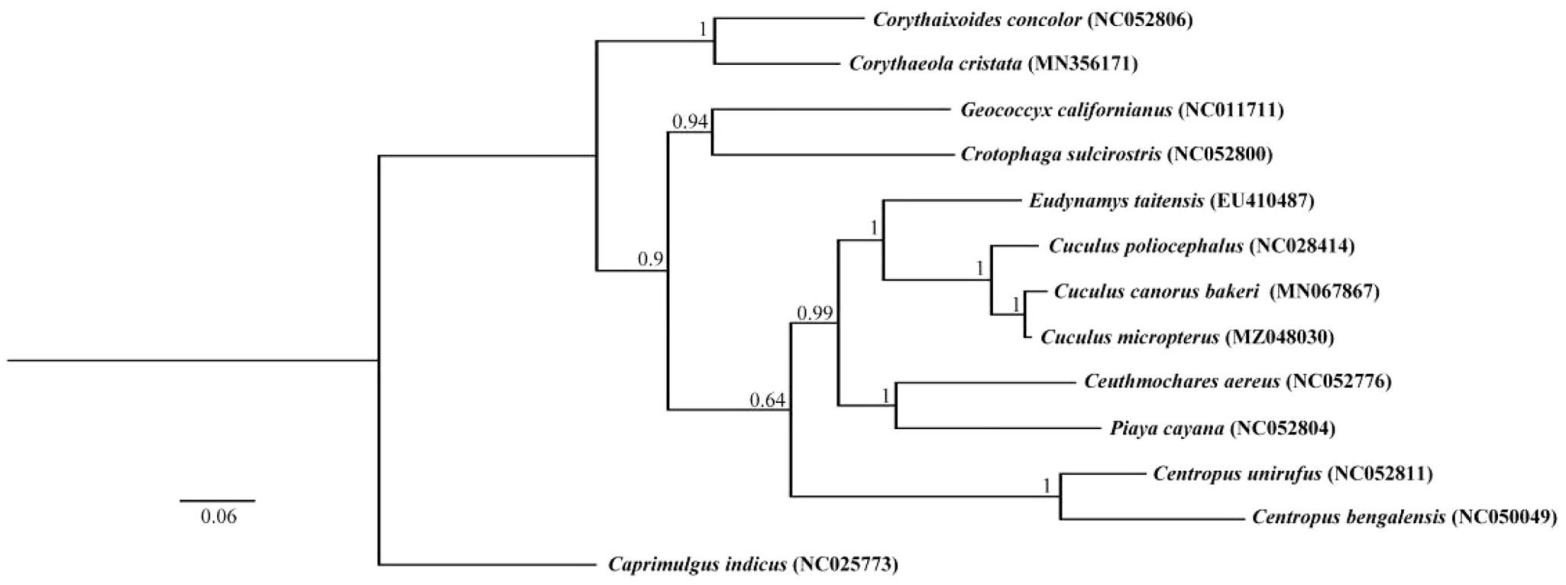
Bayesian phylogenetic analyses for *Cuculus micropterus* based on the 13 mitochondrial PCGs nucleotide sequences of 13 species.

## Data Availability

All data included in this study are available within the article and in Genbank (BankIt (nih.gov) or http://www.ncbi.nlm.nih.gov/) under the accession number MZ048030.
